# Influence of social factors on adoption of sanitation practices in rural areas: a mixed methods study in Nzaui, Kenya

**DOI:** 10.11604/pamj.2023.46.16.35770

**Published:** 2023-09-12

**Authors:** Grace Kasiva Eliud, Lilian Mukiri Kirimi, Kirema Nkanata Mburugu

**Affiliations:** 1 Sanitation Research Institute, Meru University of Science and Technology, Meru, Kenya,; 2 Department of Business Studies, University of Embu, Embu, Kenya

**Keywords:** Social factors, sanitation practices, rural areas, improved sanitation

## Abstract

**Introduction:**

provision of adequate sanitation is among the common strategies of preventing sanitation-related diseases. However, provision of sanitation facilities may only be a sustainable solution if the population´s behavior changes and positive perception is embraced. This paper highlights the influence of social factors on adoption of sanitation practices.

**Methods:**

convergent mixed methods design was employed. Quantitative data was gathered using structured questionnaires from 100 household heads selected using cluster and simple random techniques. Logistic regression analysis was performed to explore factors that influenced adoption of sanitation practices. Qualitative data was gathered from a purposively selected focus group and analyzed thematically.

**Results:**

many (57%) of the participants were males. The average age for participants was 39 years, standard deviation (SD)=0.20. From the multivariable regression analysis with adjusted odds, household heads being aged 18-33 years (OR 1.76, 95% CI: 0.62-3.02, p=0.015) and safety of latrines (OR 1.72, 95% CI: 0.70-5.15, p<0.001) was associated with increased open defecation chances; whereas being a female (OR 0.16 95% CI: 0.06-1.81, P=0.01), availability of open spaces near households (OR 0.12, 95% CI: 0.05-1.13, p=0.30), and mason skills (OR 0.29, 95% CI: 0.13-1.65) were associated with reduced likelihood of open defecation practices. Further, being a female (OR 1.06, 95% CI: 0.18-3.16, p=0.043), having knowledge on safe sanitation (OR 1.01, 95% CI: 0.74-3.08, p=0.02), engaging skilled masons for toilet construction (OR 1.299, 95% CI: 1.01-8.95, p=0.005) and financial stability (OR 1.95, 95% CI: 0.98-23.40, P=0.032<0.001) were positively associated with adoption of improved toilets.

**Conclusion:**

the sanitation status in the study area was mainly poor due to the influence of multiple factors like gender, absence of toilets, knowledge on safe sanitation, poverty, mason skills and toilet location in relation to safety. The findings showed the need for innovative planning approaches based on the social aspects of communities for progress in sanitation standards in rural areas. Such approaches should adhere to the sanitation hardware versus software components of communities to promote active utilization of the available toilets, construction of improved toilets and reduction of open defecation.

## Introduction

The sustainable development goals (SDGs) agenda 6.2 targets the achievement of basic sanitation and hygiene and an end of open defecation for all by 2030 [1] as a strategy towards prevention of sanitation-related diseases such as diarrhea [2]. Approaches instituted by the government to promote improved sanitation in rural areas such as community-led total sanitation and public health awareness creation have unexpectedly yielded poor outcomes because even with toilets, people still practice open defecation [2]. Although toilets and support may be provided, latrines adopted in developing countries, Kenya included, are at times rudimental and rural communities continue lagging behind in achieving the expected sanitation standards [3]. Defecation in the open and use of poor toilets could expose the population to diarrheal incidences responsible for 88% of deaths among children in sub-Saharan Africa [4]. Sanitation is surrounded by various issues [5], which should be addressed before providing toilet facilities else such facilities be unacceptable. This study demonstrates that social factors influence adoption of sanitation practices in rural areas thus affecting progression up the sanitation ladder.

Countries may move up the sanitation ladder when people have resources for construction of safe toilets which minimize human contact with excreta. Although the importance of safe sanitation is acknowledged, 3.6 billion people globally lack improved sanitation facilities where 14% defecate in the open with a notable access gap between developed and developing countries [3]. In countries like New Zealand, 76% of the population have safely managed sanitation facilities, 23% have attained basic sanitation services, and only 1% possess unimproved sanitation facilities while in Europe, 98% have attained improved sanitation [3]. Adoption of improved toilets in developed countries could be attributed to high investment and priority to safe sanitation facilities [6] and the scenario could be different for developing counties. Kenya as a developing country has only 33% of its population having access to improved sanitation and 9% still practice open defecation [3]. In Makueni County, Kenya, $6.38 million is lost yearly as a result of inadequate sanitation [7]. Maximizing the use and access to acceptable improved toilets could reduce the expenditures made on treatment of sanitation-related infections [5].

Approaches of increasing toilet coverage and use depend on interrelated dimensions of the hardware (facility) and behavior [8]. Sanitation facilities are likely to be more acceptable when they are safe [2] and guarantee privacy [9] and when residents have the desired knowledge on the importance of using toilets for excreta disposal [10]. In Ghana and Ethiopia, a study by [11] also established that for proper toilets to be constructed, masons needed to be equipped with the necessary skills of latrine construction. Unless the population´s behavior changes and a positive perception is embraced, rural residents could continue adopting underutilized toilets. The increasing mortality rates of children in developing countries as a result of easily preventable diseases such as diarrhea warrants urgent attention in rural sanitation where the children mostly live. Although behavioral issues could differ from region to region [5], there exist insufficient documentation on the influence of social factors on adoption of sanitation practices in rural areas which this study aimed to address.

## Methods

**Study design and setting:** this study adopted a convergent design which permitted simultaneous gathering of both qualitative and quantitative data. The study was conducted in Nzaui Sub-County of Makueni County between November 2021 and January 2022. The area is a water-stressed region predominantly inhabited by the Kamba tribe, who live in homesteads containing male household heads, their wives, children, and sometimes their children´s families.

**Definition of study variables:** sanitation practices were here in used to mean open defecation, toilet abandonment and adoption of unimproved toilets. According to [3], open defecation is the practice of leaving faeces in the open and unimproved toilets are those sanitation facilities (used here in to mean latrines or toilets) which do not completely prevent human contact with excreta. Social factors are the distinctive aspects in a society that influence people´s lifestyle and facilitate adoption of a given behavior [12]. A comprehensive definition of key variables used in the study is as contained in Table 1.

**Table 1:** definition of variables

Variable	Definition
Sanitation practices	Open defecation and adoption of unimproved toilets
Open defecation	The practice of excreting and leaving human faeces in the open or in the environment
Toilet abandonment	Failing to use toilets even when provided at the households
Improved toilets	Sanitation facilities which completely prevent human contact with excreta
Social factors	Are the distinctive aspects in a society that influence people’s lifestyle and facilitate adoption of a given behavior (the factors as used in the study include:presence/absence of toilets in the household, Knowledge on sanitation, poverty, space availability at the household, latrine safety, age and gender)

**Study population:** the study targeted household heads within Nzaui Sub-County, who were aged 18 years and above and who had stayed in their households for at least 2 years. Children and people who had lived in the area for less than 2 years were excluded. The total number of households in the study area is 30806 [13]. Members of one household share a single toilet block [14] hence the grounds for considering households. The basis of picking household heads for participation was that they took overall charge of their families and therefore they were likely to give the desired information concerning their homes. The study also engaged purposively selected public health officers, community health volunteers, a chief and masons in a focus group as they were thought to have an in-depth knowledge and information on sanitation issues at the community level.

**Sampling:** the study targeted a sample of 100 household heads calculated using Yamane´s formula [12] as shown:


$$n=N/\left(1+N\left(e^{2}\right)\right)$$

Where, n= desired sample, N=target population size, e=sampling error (taken to be ±10%) =30,806/309.06= 99.7 ≈ 100 respondents. Cluster sampling technique was employed in categorizing Nzaui Sub-County into clusters of its respective five Wards namely, Mbitini, Mulala, Nguu, Kalamba, and Matiliku [13]. Proportionate simple random technique was then used to identify household heads within the households in the clusters to ensure that all subjects, although from an unevenly distributed population, stood an equal chance of participation [15]. The distribution of samples in the study area was as illustrated in Table 2 whereby, the number of participants per cluster was obtained by dividing the number of households per cluster by the total households and the outcome multiplied by the desired sample size.

**Table 2:** distribution of samples and response rate

Ward	Number of households per cluster or ward Ns)	Sample targeted per ward (ns) =(Ns/N) ×n	Number that showed up	Percent (%)
Kalamba	4635	15	15	15
Matiliku	4884	16	16	16
Mbitini	6867	22	22	22
Mulala	8051	26	26	26
Nguu	6369	21	21	21
**Total**	Total households (N) = 30806	Desired sample size (n)=100	100	100

**Data collection:** quantitative data was collected from 100 household heads using structured questionnaires which were self-administered. The questionnaires contained information on the demographic characteristics of the population, sanitation practices and the influence of social factors like presence of toilets in the households, knowledge on sanitation, poverty, masons´ skills, space availability near households and gender roles on adoption of sanitation practices. An observation checklist was attached to each household questionnaire where the researchers observed whether toilets were present at the households, cases of faeces left in the open and the condition of the toilets provided at the households. Qualitative data was gathered using focus group discussion guide which contained open-ended questions on the influence of social factors on adoption of sanitation practices to complement the quantitative data. The focus group consisted of 9 participants who included 2 public health officers, 2 community health volunteers, 1 area chief, 2 household heads, and 2 masons who were selected based on the principle of data saturation.

**Statistical analysis:** the statistical package for social sciences (SPSS) version 25 was used in the analysis of quantitative data to generate descriptive statistics in percentages, means and standard deviation. The data was also analyzed in inferential statistics where logistic regression analysis was done to explore the factors that influenced adoption of sanitation practices. Univariable analyses were done to analyse the association between adoption of sanitation practices and each covariate in turn and findings presented as adjusted odd ratios at 95% confidence intervals (CIs). The univariable analysis was followed by multivariable analyses where the association between adoption of sanitation practices and each covariate obtained from the univariable analysis was examined. At this stage, all the significant variables from the univariable model were assessed in the multivariable model then removed step by step depending on the least significant variable until all the model variables remaining were statistically significant. The adjusted model contained covariates that had a significant association with sanitation practices and presented as ORs with 95% CIs. The indicators whose level of significance was p<0.05 were considered to be significant influencers of adoption of sanitation practices. Qualitative data was coded in the MAXQDA software and organized into themes guided by the study indicators and presented in narratives.

**Ethical considerations:** ethical approval for conducting research was obtained from the Meru University Institutional Research Ethics Review Committee (MIRERC) (Reference number: MU/1/39/28 Vol.2-32). Informed consent was obtained from every participant to ascertain willingness to participate in the study. Participants had the freedom to withdraw from participation even when the study was half way if they so wished with no consequences whatsoever. Respondents were reassured of the safety of the information gathered and that it was not to be used for any malicious reasons.

## Results

Overall, the study targeted 100 respondents from households. The response rate was 100% which qualified the data adequate for analysis and reporting. The focus group discussion participants showed up in time and responded to the discussion questions adequately. More males (57%) than females took part in the study and the average age of participants was 39 years, standard deviation (SD) =0.20, with those aged 18-33 and 34-49 years accounting for the highest percentage (38% for both). Only 2% of the respondents lacked formal education. Christianity was the predominant religion covering 98% of the sampled population. With respect to sanitation practices, the highest percentage (76%) of the population used traditional pit latrines, while other few options included improved pit latrines and flush toilets. Participants adopted unimproved toilets and practiced open defecation at means of 3.31 SD=0.32, and 2.60, SD=0.14 respectively. In the univariate model for the odds of open defecation practices (Table 3), females were 0.85 times less likely to practice open defecation compared to males (Unadjusted OR 0.85, 95% CI: 0.30-2.40, p=0.009). Decreasing age of household heads seemed to be associated with higher odds of open defecation practices compared with adults aged above 50 years (reference category). For example, participants classified as household heads and aged 18-33 years were 2.14 times more likely to defecate in the open as compared to the reference age category (OR 2.14, 95% CI: 0.65-3.39, P=0.005). People who lacked toilets in their households had 1.20 higher odds of practicing open defecation than people with toilets (OR 1.20, 95% CI: 0.52-2.78, P=0.012). Results also showed that availability of open spaces around the households appeared to lower the probability of practicing open defecation as the odds of open defecation were 61% lower where open spaces were available in the households (OR 0.39, 95% CI: 0.12-1.34, p=0.006). The odds for practicing open defecation in households using toilets constructed by skilled masons were 25% lower than those that used toilets constructed by unskilled masons (OR 0.75, 95% CI: 0.29-1.96, p=<0.001). In the adjusted model, the variables were statistically significant other than toilet absence. Particularly, the likelihood of open defecation for people aged 18-33 was higher than for those aged above 50 years (OR 1.76, 95% CI: 0.62-3.02, p=0.015). People using unsafe latrines (OR 1.72, 95% CI: 0.70-5.15, p<0.001) had increased chances of defecating in the open. Being a female (OR 0.16 95% CI: 0.06-1.81, P=0.01), availability of open spaces near households (OR 0.12, 95% CI: 0.05-1.13, p=0.30), and mason skills (OR 0.29, 95% CI: 0.13-1.65, p=<0.001) remained negatively associated with open defecation practices. In the univariate model for the odds of adoption of improved toilets (Table 3), the chances that women headed households would adopt improved toilets were higher by 2.89 than households headed by men (Unadjusted OR 2.89, 95% CI: 0.24-3.99, p=0.017). People who had knowledge on the importance of safe sanitation had 2.73 odds of adopting improved toilets compared to those who lacked knowledge (OR 2.73, 95% CI: 0.86-3.91, p=<0.001). Toilets constructed by skilled masons had higher odds of being improved compared to those constructed by unskilled masons (OR 3.67, 95% CI: 1.12-11.95, p=0.031). The financially stable households were 7.02 times likely to adopt improved toilets than their counterparts (OR 7.02, 95% CI: 1.60-30.76, p=0.01). All factors noted as significant in the unadjusted model remained significant after adjusting for other variables (p<0.05). In the multivariate model for adjusted odds, being a female (OR 1.06, 95% CI: 0.18-3.16, p=0.043), having knowledge on safe sanitation (OR 1.01, 95% CI: 0.74-3.08, p=0.02), mason skills (OR 1.299, 95% CI: 1.01-8.95, p=0.005) and financial stability (OR 1.95, 95% CI: 0.98-23.40, P=0.032) were positively associated with adoption of improved toilets.

**Table 3:** odds of adoption of sanitation practices in Nzaui Sub-County (n=100)

Variable	Open defecation practice			
**Unadjusted ORs (95% CI)**	**P-value**	**Adjusted ORs (95% CI)**	**P-value**
**Gender**				
Female	0.85 (0.30-2.40)	0.009	0.16 (0.06-1.81)	0.010
**Age, years**				
18-33	2.14 (0.65-3.39)	0.05	1.76 (0.62-3.02 )	0.015
34-49	1.39 (0.43-4.54)	0.584	0.33 (0.25-3.46)	0.668
Toilet absence	1.20 (0.52-2.78)	0.012	0.18 (0.11-2.04)	0.075
Knowledge	1.06 (0.288-3.91)	0.929	0.06 (0.04-2.39)	0.762
Availability of open spaces	0.89 (0.12-1.43)	0.006	0.12 (0.05-1.13)	0.030
Skilled masons	0.75 (0.29-1.96)	<0.001	0.29 (0.13-1.65)	<0.001
**Poverty**				
Financially stable	0.14 (0.63-3.07)	0.417	0.33 (0.70-3.98)	0.456
**Toilet location and safety**				
Far from the household	2.06 (0.70-5.59)	<0.001	1.72 (0.70-5.15)	<0.001
**Variable**	**Adoption of improved toilets**			
	**Unadjusted ORs (95% CI)**	**P-value**	**Adjusted ORs (95% CI)**	**P-value**
**Gender**				
Female	2.89 (0.24-3.99)	0.017	1.06 (0.18-3.16)	0.043
**Age, years**				
18-33	0.43 (0.06-3.01)	0.397	0.39 (0.06-2.58)	0.445
34-49	1.78 (0.34-9.31)	0.496	0.58 (0.32-9.18)	0.576
Toilet absence	0.623 (0.22-1.81)	0.383	0.473 (0.14-1.78)	0.492
Knowledge	2.73 (0.86-3.91)	<0.001	1.01 (0.74-3.08)	0.020
Availability of open spaces	0.92 (0.32-2.69)	0.882	0.08 (0.03-2.53)	0.071
Skilled masons	3.67 (1.12-11.95)	0.031	1.299 (1.01-8.95)	0.005
**Poverty**				
Financially stable	7.02 (1.60-30.76)	.010	1.95 (0.98-23.40)	0.032
**Toilet location and safety**				
Far from the household	0.38 (0.09-1.59)	0.185	0.97 (0.73-1.76)	0.504

**Qualitative results:** location of toilets far from households was associated with poor excreta disposal practices. A respondent from the focus group discussion said: “*There are many people in this community who would not accept leaving their faeces just that way, they would rather dig a small hole when the toilet is far to hide their faeces*.” Knowledge was important for behaviour change, however, it would be ineffective especially for residents with low incomes as they would struggle to build improved toilets as argued by focus group discussion respondents who said: *Even if you taught people about toilets, if they had no capacity to construct good toilets, they would still construct toilets made of sacks and polythene papers*.” “*Teaching or educating the community about toilet use was not an issue, the problem arose when the person being taught could not afford the construction materials and costs associated with its construction. Like in one village here, we organized ourselves as a community to support the construction of a simple latrine for an old poor woman*.” Focus was mostly on meeting primary needs other than secondary needs such as construction of good toilets in such a region that experienced episodes of drought. A respondent argued that: “*The County seems to be extremely dry and sometimes drought makes me and other community members budget for food only and not secondary needs like good toilets. You cannot construct good toilets and lack food*.” When asked whether open spaces around the households influenced open defecation, a participant reported that the spaces available were too open to encourage defecation: “*There are no thickets around. The open spaces available are really open, they cannot allow you to hide and relieve yourself as you will just be seen by passers-by from a distance*.” Respondents supported that training of masons was essential in the construction of improved toilets. A participant said that: “*There are trained masons but not specifically for toilet construction. Mostly the people who come to construct toilets are imported from some place, and they construct the toilets according to the directions and instructions of the head of the family. You know most of them know nothing about toilet designs*.”

## Discussion

The study identified social factors related with adoption of sanitation practices such as open defecation and use of unimproved toilets in rural areas of Nzaui, Kenya. Particularly, the factors ranged from gender, age of the household head, absence of toilets in the households, availability of open spaces around the households, skilled masons for toilet construction and knowledge on safe sanitation The association between gender and open defecation could be attributable to variations in gender roles at the households. Culturally, while women are engaged in household chores such as cooking, washing and looking after children, men take roles like cattle keeping away from households in open fields or bushes where there are no toilets [12-16] which could have facilitated higher chances of open defecation for men. The active engagement of middle-aged household heads in roles like cattle rearing compared to elderly adults, as well as the fact that age 18-33 is the age of active rearing of children who had a tendency of defecating in the open could possibly explain the increasing odds of open defecation with decreasing age of the household head. The findings agreed with the study conducted in Ethiopia [17] and in Nepal [18]. Toilets are constructed when space is available in the household. Thus, absence of enough space around the households could discourage construction of toilets therefore encouraging the practice of open defecation. Similar results were obtained in Ethiopia where researchers demonstrated the essence of open spaces around the households in encouraging toilet adoption [19]. Preference of open defecation when toilets were located far from the households could be explained by the fact that residents would feel unsafe while using the facilities which were far from their dwelling places because they were risky and could attract cases of rape (for women) or anxiety [20]. People were likely to adopt improved toilets and avoid open defecation when masons with the necessary skills were engaged for toilet construction. Better toilets could be adopted when skilled masons are engaged to construct toilets as masons with desired skills are likely to construct toilets in proper designs, acceptable to users. Similar conclusions were made in Ghana and Ethiopia where mason training on toilet construction resulted in properly designed sustainable sanitation facilities as it equipped masons with the necessary skills for erecting toilets [11]. The increased likelihood of adoption of improved toilets for households headed by women could be associated with the fact that women, unlike men, may deem improved toilets an important investment because they, together with children, are the most users of toilets and desired toilets which were safe and promoted privacy. Researchers have suggested that when men dominate decision-making over sanitation matters, it is likely that the sanitation options availed may fail to address the needs of women and children [2].

The findings underline the criticality of expanding public health collaborations with non-governmental organizations and the community to promote active surveillance and training at the households for acceptable and improved household toilets. The multi-sectoral approach is at the core of the sustainable development agenda on sanitation to ensure adoption of safe toilets at the households [2]. The direct relationship between participants´ knowledge and adoption of improved toilets could be explained by the fact that people who understood the essence of having toilets which separated human contact from excreta were unlikely to construct pathetic toilets. Such people might have understood the mechanisms of faecal transfer from unimproved toilets to their food or systems through nuisances such as flies or cockroaches. Similar results were also reported in Uganda [12]. However, knowledge alone did not encourage people to avoid the negative sanitation practice of adopting unimproved toilets but other factors including financial capabilities of residents impeded them from improving toilets. It was noted that the community was willing to use good toilets, but poverty in the area prevented the construction of standard superstructures. The process of toilet construction could have been expensive to the majority of the residents which resulted in adoption of poor latrines. The findings implied that people could adopt toilets made of poor materials, without slabs or roofs if they could not afford the costs associated with construction of improved sanitation facilities. Similar results were obtained in Ghana where increased poverty levels spearheaded increased adoption of rudimentary toilets [21]. Thus, there is need of designing improved toilets with locally available and cheap construction materials in rural areas to favor the poor.

A reliable and honest feedback was essential. Researchers had to explain the concept of sanitation before engaging participants in the study to eliminate response bias. Besides, some respondents might have reported what was generally viewed as correct and acceptable by the community and not what they deemed to be true themselves which was beyond the researchers´ capacity to control. In addition, the population sample might limit generalizability of findings. Therefore, the way social factors influence adoption of sanitation practices might not be completely applicable for all rural areas and may require further exploration into the generalization of these factors. The study extended knowledge in research on adoption of sanitation practices in rural areas.

## Conclusion

The sanitation status in the study area was mainly poor due to the influence of multiple factors like gender, age, absence of toilets in the household, knowledge, poverty, skills and toilet location in relation to safety. The findings thus underline the need for innovative approaches of planning based on communities´ social aspects to promote progress in sanitation standards in rural areas. The approaches should be adherent to the sanitation hardware versus software components of communities to ensure active latrine utilization, construction of improved toilets and reduction of open defecation

### What is known about this topic

- *Residents in rural areas of developing countries continue to stagnate in the sanitation ladder;*
- *Efforts to improve sanitation standards in rural areas are unexpectedly yielding poor outcomes*.

### What this study adds

- *The study has identified social factors related to adoption of sanitation practices in rural areas;*
- *The study re-emphasizes the essence of behavior change strategies that are context-specific to minimize slippage, adoption of unimproved toilets and avoidance of available toilets;*
- *This study yields recommendations applicable in promoting behavior change in rural areas*.
